# Prevention and management of hearing loss in patients receiving ototoxic medications

**DOI:** 10.2471/BLT.21.286823

**Published:** 2022-09-02

**Authors:** Michael M Lindeborg, David H Jung, Dylan K Chan, Carole D Mitnick

**Affiliations:** a Department of Otolaryngology, University of California, San Francisco, United States of America (USA).; b Department of Otolaryngology, Harvard Medical School, Boston, USA.; c Department of Global Health and Social Medicine, Harvard Medical School, 641 Huntington Ave, Boston, Massachusetts 02115, USA.

## Abstract

Following the efforts of patient advocates, the World Health Organization published updated guidelines for management of multidrug-resistant tuberculosis in 2018 that advised against the routine use of ototoxic second-line injectable drugs (amikacin, capreomycin and kanamycin). Although hearing loss is no longer considered an unavoidable harm for patients with multidrug-resistant tuberculosis, ototoxic medications continue to be used for several infectious and oncological disorders around the world. These drugs contribute to more than a half a million cases of hearing loss worldwide annually. Currently, there are no international standards for preventing and managing hearing loss associated with ototoxic medications. We present recent data on the prevention and management of hearing loss related to these drugs and highlight the variability in care across settings. More importantly, we aim to provide an evidence-based framework for evaluating, screening and preventing ototoxicity. Finally, we identify avenues for future research so that patients no longer have to choose between hearing loss and a disease cure. There remain significant gaps in our understanding about optimal screening and treatment of ototoxic hearing loss. Here we aim to inspire future international guidelines to address gaps in ototoxicity care and establish research agendas for eliminating ototoxic medications.

## Résumé

Sous l'impulsion des défenseurs des droits des patients, l'Organisation mondiale de la Santé a publié une version actualisée des lignes directrices relatives à la prise en charge de la tuberculose multirésistante en 2018, qui déconseille l'usage systématique de médicaments ototoxiques injectables de deuxième intention (amikacine, capréomycine et kanamycine). Bien que la perte auditive ne soit plus considérée comme un risque inévitable chez les patients atteints de tuberculose multirésistante, les médicaments ototoxiques continuent à être largement employés pour traiter de nombreuses maladies infectieuses et oncologiques à travers le monde. Ces médicaments sont impliqués chaque année dans plus de la moitié des cas de déficience auditive dans le monde. Il n'existe actuellement aucune norme internationale consacrée à la prévention et à la prise en charge de la perte auditive causée par des médicaments ototoxiques. Dans le présent document, nous exposons les données récentes à ce propos et soulignons la variabilité des soins prodigués d'une région à l'autre. Nous tentons surtout d'établir, à partir d'éléments concrets, un cadre dédié à l'évaluation, au dépistage et à la prévention de l'ototoxicité. Enfin, nous dégageons des pistes pour de futures études, afin que les patients n'aient plus à choisir entre une perte auditive et un remède. D'importantes lacunes subsistent dans notre compréhension du dépistage et du traitement de la perte auditive d'origine ototoxique. Nous espérons inspirer de futures lignes directrices internationales afin d'y remédier et de développer des programmes de recherche pour supprimer les médicaments ototoxiques.

## Resumen

Tras los esfuerzos de los defensores de pacientes, la Organización Mundial de la Salud publicó en 2018 unas directrices actualizadas para el tratamiento de la tuberculosis multirresistente en las que se desaconsejaba el uso rutinario de medicamentos inyectables de segunda línea ototóxicos (amikacina, capreomicina y kanamicina). Aunque la pérdida de audición ya no se considera un daño inevitable para los pacientes con tuberculosis multirresistente, los medicamentos ototóxicos se siguen administrando para varios trastornos infecciosos y oncológicos en todo el mundo. Estos fármacos contribuyen a más de medio millón de casos de pérdida de audición en todo el mundo cada año. En la actualidad, no existen estándares internacionales para prevenir y tratar la pérdida de audición asociada a los medicamentos ototóxicos. En este documento, se presentan datos recientes sobre la prevención y el tratamiento de la pérdida de audición relacionada con estos fármacos y se destaca la variabilidad de la atención en los distintos entornos. Además, se pretende ofrecer un marco basado en la evidencia para evaluar, detectar y prevenir la ototoxicidad. Por último, se identifican las vías de investigación futura para que los pacientes no tengan que elegir entre la pérdida de audición y la cura de la enfermedad. Siguen existiendo importantes deficiencias en el conocimiento del cribado y el tratamiento óptimos de la pérdida de audición ototóxica. En este sentido, se pretende inspirar futuras directrices internacionales para abordar las deficiencias en la atención a la ototoxicidad y establecer programas de investigación para eliminar los medicamentos ototóxicos.

## ملخص

بعد الجهود التي بذلها المدافعون عن المرضى، نشرت منظمة الصحة العالمية إرشادات محدثة لإدارة السل المقاوم للأدوية المتعددة في عام 2018، والتي نصحت بعدم الاستخدام الروتيني لعقاقير تسمم الأذن من المستوى الثاني القابلة للحقن (أميكاسين، وكابريوميسين، وكاناميسين). على الرغم من أن فقدان السمع لم يعد ضررًا لا مفر منه للمرضى الذين يعانون من مرض السل المقاوم للأدوية المتعددة، إلا أن أدوية تسمم الأذن لا تزال تُستخدم للعديد من الاضطرابات المعدية والأورام في جميع أنحاء العالم. تساهم هذه الأدوية في أكثر من نصف مليون حالة فقدان للسمع في أنحاء العالم سنويًا. وفي الوقت الحالي، لا توجد معايير دولية لمنع فقدان السمع المرتبط بأدوية تسمم الأذن، وعلاجه. نحن نقدم بيانات حديثة حول الوقاية من فقدان السمع وعلاجه، فيما يتعلق بهذه العقاقير، كما نركز على التباين في الرعاية عبر البيئات المختلفة. والأكثر أهمية من ذلك، نحن نهدف إلى توفير إطار عمل قائم على الأدلة لتقييم وفحص والوقاية من تسمم الأذن. وأخيرًا، نحن نحدد وسائلاً للبحث في المستقبل حتى لا يضطر المرضى للاختيار بين فقدان السمع وعلاج المرض. لا تزال هناك فجوات واضحة في استيعابنا للفحص والعلاج الأمثل لفقدان السمع السام. نحن هنا نهدف إلى بث الإرشادات التوجيهية الدولية المستقبلية للتعامل مع الفجوات في رعاية تسمم الأذن، ووضع جداول أعمال للبحث للتخلص من أدوية تسمم الأذن.

## 摘要

在患者倡导者的努力下，世界卫生组织于 2018 年发布了最新的耐多药结核病管理指南，建议不要常规使用耳毒性二线注射药物（阿米卡星、卷曲霉素和卡那霉素）。尽管听力损伤不再被认为是对耐多药结核病患者无法避免的伤害，但耳毒性药物仍在世界各地用于治疗几种传染性和肿瘤性疾病。这些药物每年导致全球超过 50 万例患者听力损伤。目前，还没有一个国际标准来预防和管理与耳毒性药物相关的听力损伤。我们提供了与这些药物相关的听力损伤预防和管理的最新数据，并强调了不同环境下护理的差异。更重要的是，我们的目的是为评估、筛查和预防耳毒性提供一个基于证据的框架。最后，我们确定了未来研究的途径，使患者不再需要在听力损伤和疾病治愈之间做出选择。我们对耳毒性听力损伤的最佳筛查和治疗的理解仍然存在重大差距。在此，我们的目标是激发未来的国际指南，以解决耳毒性护理方面的差距，并制定消除耳毒性药物的研究议程。

## Резюме

Благодаря усилиям активистов по защите интересов пациентов в 2018 году Всемирная организация здравоохранения опубликовала обновленное руководство по ведению туберкулеза со множественной лекарственной устойчивостью, в которой рекомендовалось воздержаться от обычного использования ототоксичных инъекционных препаратов второй линии (амикацин, капреомицин и канамицин). Хотя потеря слуха больше не считается неизбежным вредом для больных туберкулезом со множественной лекарственной устойчивостью, ототоксичные препараты продолжают использоваться для лечения ряда инфекционных и онкологических заболеваний во всем мире. Эти препараты ежегодно вызывают более полумиллиона случаев потери слуха во всем мире. В настоящее время не существует международных стандартов по профилактике и лечению потери слуха, связанной с применением ототоксичных препаратов. Авторы презентуют свежие данные о профилактике и лечении потери слуха, связанной с данными препаратами, и подчеркивают различия в лечении в разных условиях. Что еще более важно, авторы стремятся предоставить основу для оценки, скрининга и профилактики ототоксичности с учетом накопленных фактических данных. Наконец, авторы определяют направления будущих исследований, чтобы пациентам больше не приходилось выбирать между потерей слуха и лечением болезни. В понимании авторов в отношении оптимального скрининга и лечения потери слуха из-за ототоксичности остаются значительные пробелы. В данной статье они стремятся вдохновить коллег на разработку будущих международных руководств по устранению пробелов в лечении ототоксичности и исследовательских программ по исключению ототоксичных препаратов.

## Introduction

Before 2018, second-line injectable drugs were core components of multidrug-resistant tuberculosis regimens.^1^ Major international treatment guidelines recommended their use despite weak in vitro evidence and conflicting results from retrospective cohort studies.^2^^,^^3^ Importantly, a review of ototoxicity associated with second-line injectable drugs reported irreversible hearing loss in up to 50% of exposed patients.^4^ With the introduction of novel antibiotics such as bedaquiline and delamanid, and the repurposing of antimicrobials such as linezolid and clofazimine, new options emerged for multidrug-resistant tuberculosis treatment. Ultimately, driven by years of advocacy by patients around the world, second-line injectable drugs fell out of favour.^5^^,^^6^

In 2018, this movement culminated in updated multidrug-resistant tuberculosis treatment guidelines by the World Health Organization (WHO). The guidelines, updated in 2020, advised against the use of second-line injectable drugs, kanamycin and capreomycin, due to the increased risk of treatment failure, relapse and severe side-effects.^7^ Amikacin and streptomycin, newly classified as second-line drugs, are now recommended only after exhausting other treatment options and when audiometry monitoring is available.^7^ Similar guidance was released by the American Thoracic Society, European Respiratory Society and Infectious Diseases Society of America in 2019.^8^ Overall, this movement reaffirmed patients’ right to hearing and demonstrated that advocacy and research can lead to effective treatment alternatives.

However, patients continue to receive ototoxic medications for certain infectious diseases, cancers and chronic health conditions (Table 1). Globally, platinum-based chemotherapies and aminoglycosides are associated with over 500 000 and 50 000 annual cases of hearing loss, respectively.^4^^,^^15^ Currently, there are no international guidelines or protocols for the screening and management of hearing loss during treatment.^7^ Examination of several countries’ guidance for detection of ototoxicity shows that there is no universal approach to screening or diagnosing hearing loss.

**Table 1:** Ototoxicity by drug class

Drug class	Mechanism of ototoxicity	Type	Incidence of ototoxicity^a^
Aminoglycosides	Outer hair cells	Irreversible	40.6% (95% CI: 32.8–66.6%) of 1386 patients treated^9^
Platinum-based compounds	Outer hair cells, stria vascularis, spiral ganglion	Irreversible	Cisplatin: 49.2% (95% CI: 42.6–55.8%) of 3224 patients treated^10^ Carboplatin: 13.5% (95% CI: 8.7–20.3%) of 742 patients treated
Loop diuretics	Sodium, potassium and chloride channel	Reversible	3.3% (29/878) of patients treated^11^
Macrolides	Stria vascularis	Reversible	50–100 total cases up to year 2003^12^
Vancomycin	Unknown	Reversible	8% (7/92) of patients treated^13^
Salicylates, nonsteroidal anti-inflammatory drugs	Vasoconstriction in stria vascularis	Reversible	250 cases per 19 832 person-years^14^

CI: confidence interval.

^a^ Incidence of ototoxicity is based on reported proportions in retrospective cohort studies. The evidence was not strong, as the reported incidence of ototoxicity was widely variable and higher incidence was associated with higher doses or prolonged exposure. The 95% CI are from available meta-analyses with pooled data.

Standardized guidance and research are needed, especially in low-resource settings, to detect and treat hearing loss from ototoxicity. Here, we propose an approach to early detection and management of ototoxic hearing loss, provide evidence-based treatment options and identify avenues for future research.

## Ototoxic drugs 

Commonly used ototoxic drugs, and their incidence and mechanisms of ototoxicity are summarized in Table 1.

### Ototoxic antibiotics

Aminoglycosides are an injectable antibiotic class with a reported irreversible ototoxicity of 3–50%.^16^ Ototoxic effects occur in a dose-dependent fashion via reactive oxygen species generated in the patient’s cochlear hair cells. Given the lower cost and fewer supply-chain barriers, aminoglycosides continue to be regularly used in low- and middle-income countries, as illustrated by a case study in Peru (Box 1). Additionally, there are certain infectious diseases for which no effective alternative has been developed (such as *Pseudomonas aeruginosa* commonly isolated in cystic fibrosis patients, tularaemia and multidrug-resistant urinary tract infections).

Box 1 **Case study: transitioning away from second-line injectable drugs for multidrug-resistant tuberculosis in Peru**

Peru is a global leader in its efforts to combat multidrug-resistant tuberculosis. In 1994, a sampling study showed 12 out of 22 (54.4%) tested tuberculosis cases in Peru had antibiotic resistance.^17^ Through community-based treatment, Peru effectively created universal access to treatment for multidrug-resistant tuberculosis. Most recently, the health ministry reported that 139 (7.3%) of 1908 newly diagnosed cases and 44 (16.2%) of 272 previously treated patients have multidrug-resistant tuberculosis.^18^

Up until 2018, Peru included ototoxic second-line injectable drugs in their recommended regimen. With newly published guidelines from the World Health Organization^19^ and participation in novel multidrug-resistant tuberculosis randomized controlled trials (the EndTB and EndTB-Q trials),^20^ Peru has begun to incorporate new drugs such as bedaquiline and delamanid. Peru has also repurposed drugs such as linezolid and clofazimine in its shift away from second-line injectable drugs. Still, challenges remain in eliminating these drugs from standard regimens.

Second-line injectable drugs are cheaper to purchase than some newer and repurposed drugs, although monitoring and managing toxicity caused by second-line injectable drugs is costly. Physicians’ fear of generating resistance to novel drug agents also limits the shift away from second-line injectable drugs. Many low- and middle-income countries face similar challenges.

To decrease the use of ototoxic antibiotics, future research is needed to find novel antibiotic alternatives. For multidrug-resistant tuberculosis, the move away from ototoxic medications has been aided by the introduction of bedaquiline and delamanid, which are oral antibiotics with fewer associated side-effects. Parallel efforts are needed for other infectious diseases. Increasing multilateral organization support and government subsidies to low- and middle-income countries are key to accelerating this change.

Vancomycin and macrolides are two other antibiotic classes that have been associated with reversible ototoxic hearing loss, although the reported incidence is much lower compared with aminoglycosides. Whether vancomycin is directly responsible for hearing loss is contested in the literature, given that many patients are concurrently receiving other ototoxic medications (such as aminoglycosides or diuretics).^21^^–^^23^ Animal studies have demonstrated that vancomycin alone does not cause ototoxicity but may potentiate hearing loss from aminoglycosides.^24^ Macrolides have been found to be associated with sensorineural hearing loss, although the evidence is limited to case reports and focuses most commonly on systemic erythromycin. A review found that as of 2003, only 50–100 cases of macrolide-associated ototoxicity had been published.^25^^,^^26^ Risk factors associated with purported ototoxicity from vancomycin and macrolides include old age, kidney dysfunction and co-administration with other ototoxic agents.^22^ Given the relatively low incidence of ototoxicity and weak evidence that they are causative in the first place, further investigation is needed before moving away from these antibiotics.

### Platinum-based chemotherapy

Platinum-based chemotherapies, such as cisplatin, have long been associated with irreversible hearing loss. Their mechanism for ototoxicity directly affects the stria vascularis, spiral ganglion cells and hair cells of the cochlea (cisplatin is thought to preferentially affect the outer hair cells, whereas carboplatin damages the inner hair cells).^27^ Similar to aminoglycosides, hearing loss first occurs in the high frequencies of sound and progresses towards lower frequencies with prolonged medication exposure. Recent evidence suggests that cisplatin is retained in the cochlea for months to years after finishing treatment, underlying the importance of post-treatment screening.^28^ The incidence of hearing loss associated with carboplatin is estimated to be approximately 13.8% (95% confidence interval, CI: 8.7–20.3%).^10^ For cisplatin, conventional regimens are associated with a 49.2% (95% CI: 42.6–55.8%) incidence of hearing loss, and combination therapy with cisplatin is associated with a 56.1% (95% CI: 45.1–66.4%) incidence of hearing loss.^10^^,^^15^ The guidelines of the National Cancer Institute Common Terminology Criteria for Adverse Events are commonly applied when investigating ototoxicity for platinum-based compounds. However, the guidelines do not include high frequency hearing loss and may lead to underestimation of the true incidence of hearing loss.^29^ Platinum compounds are the principal treatment for several paediatric malignancies, therefore their overall impact on speech and language development in the setting of hearing loss is important.^30^^,^^31^

### Other drugs 

Loop diuretics, salicylates and nonsteroidal anti-inflammatory drugs are all associated with reversible hearing loss, and their associated incidence of hearing loss is much lower compared with aminoglycosides and platinum-based chemotherapies. Loop diuretics are thought to act on the sodium, potassium and chloride cotransporter in the cochlea and on potassium levels in the endolymph. The mechanism by which salicylates and nonsteroidal anti-inflammatory drugs affect hearing is incompletely understood, but may be related to vasoconstriction with decreased blood supply to the stria vascularis of the cochlea. Approximately 7 per 1000 individuals and 11 per 1000 individuals experience ototoxicity while on diuretics and salicylates, respectively.^25^ Of note, incidence of ototoxicity is associated with concomitant use of other ototoxic drugs.^32^ Given the widespread use of these drugs and relatively low incidence of associated ototoxicity, efforts should focus on minimizing the concurrent use of other ototoxic agents, avoiding supra-therapeutic dosing and immediately stopping their use if hearing loss is determined.^33^

## Monitoring

### Irreversible ototoxic medications

Patients who receive ototoxic medications with irreversible effects (such as aminoglycosides or platinum-based chemotherapies) should be monitored closely with audiometry to assess hearing loss. Currently, there are no universal or even comparable standards or guidelines for monitoring for ototoxic hearing loss.^34^^,^^35^ Practical country guidance recommends screening at frequencies less than 10 000 Hz, with follow-up screening every 2–4 weeks, or as soon as a patient experiences subjective hearing loss (Table 2; available at: https://www.who.int/publications/journals/bulletin/). Given that the earliest stages of ototoxic hearing loss occur at high frequencies that patients may not be aware of, we believe that screening programmes should aim to provide standard screening over a set frequency range and at specific time intervals.

**Table 2:** Variability in audiogram-based hearing loss protocols for ototoxic drugs by country

Country	Monitoring schedule	Sound frequencies monitored, Hz	Threshold shift
Brazil^36^	Baseline test, then weekly audiogram monitoring	500 to 4000	≥ 15 dB in at least two frequencies
India^37^^,^^38^	Baseline test, then test every 2 months, then test at 3 and 6 months post-treatment	250 to 8000	(i) 20 dB or greater decrease at any one test frequency; (ii) 10 dB or greater decrease at any two adjacent frequencies; or (iii) loss of response at three consecutive frequencies where responses were previously obtained
Portugal^39^	Baseline test < 72 hours after first dose, then weekly evaluation, then test at 3 and 6 months post-treatment	250 to 20 000	(i) 20 dB or greater decrease at any one test frequency; (ii) 10 dB or greater decrease at any two adjacent frequencies; or (iii) loss of response at three consecutive frequencies where responses were previously obtained
South Africa^40^^–^^42^	Wide variability. Most commonly, baseline test then monthly audiogram monitoring, then one test post-treatment	250 to 10 000	(i) 20 dB or greater decrease at any one test frequency; (ii) 10 dB or greater decrease at any two adjacent frequencies; or (iii) loss of response at three consecutive frequencies where responses were previously obtained
United States (American Speech-Language-Hearing Association)^43^	Baseline test, weekly audiogram monitoring, then test at 3 and 6 months post-treatment	250 to 20 000	(i) 20 dB or greater decrease at any one test frequency; (ii) 10 dB or greater decrease at any two adjacent frequencies; or (iii) loss of response at three consecutive frequencies where responses were previously obtained
United States-based protocol with improved feasibility^44^	Baseline test, then test after each treatment, then test at 1 and 6 months post-treatment	Sensitive range for ototoxicity	(i) 20 dB or greater decrease at any one test frequency; (ii) 10 dB or greater decrease at any two adjacent frequencies; or (iii) loss of response at three consecutive frequencies where responses were previously obtained

dB: decibel.

Note: Protocols focus on audiometry reporting and are not specific to anti-tuberculosis ototoxic medications.

**Fig. 1:**
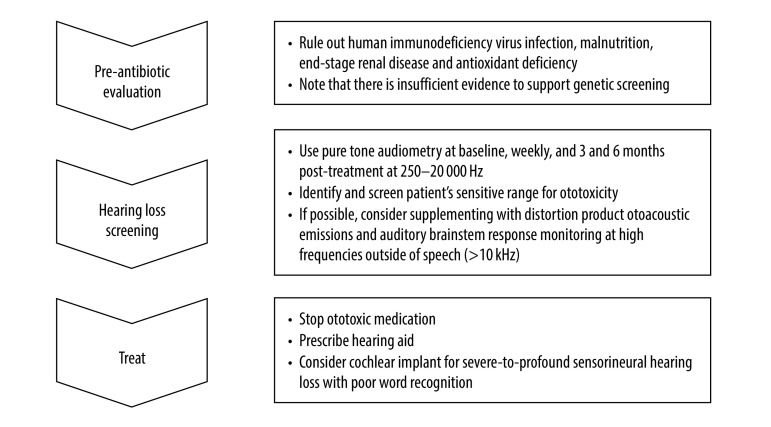
An approach to early detection and management of ototoxic hearing loss

Source: Based on expert guidance from the American Speech-Language-Hearing Association and American Academy of Audiology.^43^^,^^45^

Our suggested screening protocol for early detection and management of ototoxic hearing loss in both high-income and low- and middle-income countries is based on expert guidance from the American Speech-Language-Hearing Association and American Academy of Audiology (Fig. 1).^43^^,^^45^ We have added the sensitive range for ototoxicity, preoperative evaluation and treatment options. This guidance represents an improvement over many national guidelines because it includes high-frequency monitoring, standardizes the hearing loss threshold for ototoxicity and defines a robust monitoring schedule (Table 2).

Hearing screening for ototoxic hearing loss should monitor frequencies between 250–8000 Hz, which span the frequencies for speech.^43^ The American Speech-Language-Hearing Association recommends a baseline measurement, followed by weekly testing during therapy, and at 3 and 6 months after stopping treatment. The Association defined ototoxic hearing loss as any of the following: (i) ≥ 20 decibels (dB) change at a single frequency; (ii) ≥ 10 dB change at two or more consecutive frequencies; or (iii) loss of response at three consecutive frequencies.^43^ Monitoring frequency can be adjusted for populations who are especially susceptible to ototoxicity, including those with underlying kidney disease, malnutrition or immunodeficiencies.^46^

If countries have the capacity to expand hearing screening beyond the standard frequencies for speech, monitoring of ultra-high frequency thresholds (> 8000 Hz) may improve early detection since high frequencies are the first affected in ototoxic hearing loss. Countries such as Portugal, South Africa and the United States of America already include ultra-high frequency screening in their ototoxicity screening protocols, although the upper limit of screened frequencies varies from 10 000 to 20 000 Hz (Table 2). However, there are many countries that do not routinely screen using the full spectrum of speech frequencies or ultra-high frequency thresholds due to resource constraints. To improve the efficiency of hearing screening, the American Academy of Audiology recently proposed serial monitoring at an individual’s sensitive range for ototoxicity, which is the highest frequency with a threshold at or below 100 dB sound pressure level followed by the next six lower adjacent frequencies in one-sixth octave steps.^45^ Follow-up studies found that this method significantly outperformed conventional frequency testing in detecting emergent hearing loss, and also avoided the challenges inherent to screening with ultra-high frequencies.^47^ Implementing protocols that test for the sensitive range for ototoxicity may help achieve a higher standard of audiology care in low-resource settings where high frequency screenings were not previously possible.

Using the earliest sign of hearing threshold shift as an indication to consider stopping the ototoxic agent is essential to minimizing drug ototoxicity.^43^ This is common practice across many ototoxicity screening protocols in both high-income and low- and middle-income country settings (Table 2). When performing ultra-high frequency monitoring, the first threshold shift will likely occur above 8000 Hz. If detected at this stage, a clinician can weigh other possible treatments before ototoxic damage occurs at the lower frequencies essential for speech.^43^ In South Africa, for example, this approach has proven effective in preventing severe ototoxicity among multidrug-resistant tuberculosis patients receiving second-line injectable drugs, while maintaining relatively high treatment success.^40^^,^^48^ Although stopping treatment may reduce the degree of hearing loss compared with continuing the ototoxic agent, it does not guarantee that no further loss will occur. Unfortunately, ototoxic effects may progress for up to 6 months after stopping an injectable drug.^37^ Similar to what is found in many other national protocols (Table 2), the American Speech-Language-Hearing Association recommendation to screen at 3 and 6 months after terminating ototoxic drugs allows delayed hearing loss to be detected.

In practice, researchers – especially those from low- and middle-income countries – report difficulty in properly implementing screening protocols, and have identified gaps in audiological surveillance.^41^^,^^49^^–^^51^ This may be due to the limited resources available and the time-intensive process required to complete a comprehensive hearing screen. Some help has come in the form of the recent expansion of mobile technologies that can be used for ototoxic hearing loss screening, including SHOEBOX (Shoebox Ltd, Ottawa, Canada) and hearScreen (HearX Group Ltd, Delaware, USA).^52^^–^^55^ Many of these tools are compatible with smart devices such as smartphones or tablet computers and have the potential to be broadly implemented in low-resource settings. To conform with American Academy of Audiology guidelines, many of these tools can be programmed to perform pure tone audiometry within the normal range of speaking (250–8000 Hz) and to screen at frequencies above 8000 Hz to identify and monitor a patient’s sensitive range for ototoxicity. Additionally, though still in the early stages of development, decision-support algorithms such as OtoCalc^56^ can be implemented to help clinicians interpret audiology results and make adjustments to ototoxic medications.

Therapeutic drug monitoring should also be integrated into clinical care to provide complementary information about plasma concentrations of ototoxic agents. This information can be used to titrate doses to achieve, and not exceed, the optimal plasma concentration. This technique could mitigate any dose-related aspects of ototoxic hearing loss and other associated side-effects such as balance function.^57^^,^^58^

### Reversible ototoxic medications

For patients receiving ototoxic medications with reversible side-effects (such as loop diuretics, macrolides, vancomycin, salicylates or nonsteroidal anti-inflammatory drugs), patients need not be subjected to routine hearing screening unless they experience subjective changes in hearing, tinnitus or balance function. If patients experience any of these changes, referral for audiometric testing is appropriate. There may be a role for baseline audiograms in patients with prolonged or indefinite exposure to these medications. Future research is needed to determine what duration of time qualifies as prolonged drug exposure.

## Treatment

Hearing aids and hearing assistive technology serve as the first-line treatment for ototoxicity-induced hearing loss.^49^ These interventions merely amplify sound. Most devices are effective for sounds below 4000 Hz (speech ranges from 500–2000 Hz), but may not address the high-frequency hearing loss associated with the early stages of injectable-related ototoxicity.

Cochlear implants can improve hearing in certain patients with severe ototoxicity. Cochlear implants are indicated in individuals who have mild or severe hearing loss and poor word recognition.^34^ Use of the devices requires proper otology evaluation and device monitoring; efforts should be made to expand their application in low-resource settings. Cochlear implants have been shown to successfully restore hearing in patients with drug-associated ototoxicity.^59^^,^^60^ However, some reports of cochlear implants used for irreversible ototoxicity have identified poorer than expected performance. This outcome may be due to damage to structures beyond the hair cells, such as the first-order neurons within the spiral ganglion.^61^

## Future research

Efforts to improve the feasibility of hearing screening, especially in low-resource settings, have been explored. Suggested protocols, which have yet to be validated, recommend focusing on the sensitive range for ototoxicity only and decreasing the screening frequency to monthly tests, and one test after treatment ends.^44^^,^^62^ To overcome the complexity of robust screening protocols, future operational and quality improvement research is needed to compare and validate the performance characteristics of simplified protocols.

The impact of ultra-high frequency hearing loss (> 8000 Hz) is unclear given that most sounds of daily life occur below this frequency. Future research should investigate whether hearing loss observed at these higher frequencies indicates degeneration at the lower frequencies important for speech, and whether the medication responsible should be stopped after detection of such ultra-high-frequency hearing loss. Before clinical recommendations are established, other methods with a higher sensitivity to detect hearing loss ‒ such as otoacoustic emissions and auditory brainstem testing ‒ require further validation in subjects receiving ototoxic drugs.^47^^,^^63^ Additionally, prospective research that includes therapeutic drug monitoring is needed to balance efficacy and safety of ototoxic medications.^57^^,^^58^ Future studies should explore ways to incorporate and combine these other monitoring techniques into standardized protocols.

For irreversible ototoxic agents there should be a focus on finding alternative chemotherapies, as well as developing strategies to mitigate their consequences. Recent research efforts have investigated the use of otoprotectants such as thiosulfates, antioxidants or iron chelators (such as N-acetylcysteine, deferoxamine) to neutralize reactive oxygen species and prevent hearing loss from aminoglycosides and platinum-based chemotherapies. There continues to be mixed evidence on whether these agents are effective.^64^^,^^65^ Additionally, recent research has implicated a mechanotransducer channel as a mediator for drug entry into hair cells. Efforts to therapeutically block the mechanotransducer channel or alter the drug structure to minimize hair-cell entry are under development.^66^ Limited evidence from studies on animals suggests that intratympanic steroids may reduce ototoxicity.^67^ However, with no clinical studies at this time, there is insufficient evidence to support the routine use of these agents during the use of injectable drugs. Future research is needed to explore how hearing loss can be treated or mitigated when irreversible ototoxic agents cannot be stopped.

Hearing loss dramatically impacts quality of life across all age groups, and is associated with an increased burden of mental illness and dementia.^68^ The primary reason that patients continue to receive ototoxic medications is because there are no treatment alternatives or that access to these alternatives is limited. However, new and repurposed medications may present effective alternatives. Hearing screening would then become more robust, and information from therapeutic drug monitoring incorporated into dosing, so that patients should no longer face such a difficult choice. When these medications are deployed, resources must be made available to deal with their long-term consequences.

At the population level, avoiding and treating hearing loss is an excellent investment. Overall, the cost of hearing loss to the health-care sector, for adults and children, is estimated to range from 67 billion to 107 billion United States dollars (US$).^69^ Adults who acquire permanent hearing impairment due to ototoxic medications are estimated to have up to US$ 300 000 in medical costs. For paediatric patients who acquire prelingual hearing loss, costs can exceed US$ 1 million. Loss of productivity due to hearing loss and other costs to society from social isolation, communication difficulties and stigma are thought to add even greater costs each year.^70^ While cochlear implants are often considered prohibitively costly in settings with high burdens of tuberculosis, their cost is only US$ 77 240 per person, far less than the costs of untreated hearing loss as reported above.^71^

## Conclusions

New treatment options and revised guidelines will protect millions of people from unnecessary exposure to drugs that cause irreversible hearing loss. In the case of tuberculosis, second-line injectable drugs should be used as a last resort and should be stopped if other medication options are available. When agents that result in irreversible hearing loss are the only option, evaluation and adoption of internationally recognized guidelines are needed, alongside additional research, to prevent, manage and treat this unfortunate adverse event.
